# Multicenter Genomic Analysis of Carbapenem-Resistant Klebsiella pneumoniae from Bacteremia in China

**DOI:** 10.1128/spectrum.02290-21

**Published:** 2022-03-01

**Authors:** Astrid V. Cienfuegos-Gallet, Ying Zhou, Wenxiu Ai, Barry N. Kreiswirth, Fangyou Yu, Liang Chen

**Affiliations:** a Center for Discovery and Innovation, Hackensack Meridian Health, Nutley, New Jersey, USA; b Research Group in Basic and Applied Microbiology (MICROBA), School of Microbiology, University of Antioquia, Medellín, Colombia; c Department of Clinical Laboratory, Shanghai Pulmonary Hospital, Tongji University School of Medicine, Shanghai, China; d Department of Respiratory Medicine, The First Affiliated Hospital of Wenzhou Medical University, Wenzhou, People’s Republic of China; e Department of Medical Sciences, Hackensack Meridian School of Medicine, Nutley, New Jersey, USA; INTHERES

**Keywords:** *Klebsiella pneumoniae*, carbapenem-resistance, bacteremia, intra-hospital transmission

## Abstract

Klebsiella pneumoniae is one of the most common Gram-negative bacilli isolated from bloodstream infections worldwide, and recently an increased rate of carbapenem resistance has been reported in this pathogen. This study aims to describe the genomic characteristics of carbapenem-resistant K. pneumoniae (CRKP) isolated from patients with bacteremia in China. We analyzed 147 isolates from patients with bacteremia attended in 12 referral hospitals in China between April 2015 and November 2018. We conducted a phenotypic susceptibility evaluation and whole genome sequence analysis to characterize antimicrobial resistance profile, virulence genes, and dominant clones among CRKP. ST11 accounted for most infections (*n* = 98, 66.6%), followed by ST45 (*n* = 12, 8.2%), ST15 and ST290 (*n* = 8, 5.4% each). KPC (*n* = 98, 66.7%) and NDM (*n* = 27, 18.4%) are the main carbapenemases detected in the CRKP isolates. We detected yersiniabactin (*n* = 123, 83.7%) and aerobactin (49.9%) siderophores, and both *rmpA* and aerobactin genes in 21 ST11 isolates (21.43%), which are considered characteristic biomarkers of hypervirulent strains. Isolates showed high resistance rates to the β-lactams (>90%) and other antibiotics classes such as fluoroquinolones, aminoglycosides and tetracyclines (50%), but were susceptible to ceftazidime-avibactam (74.8%). In addition, we detected intra-hospital transmission of ST11 and ST45 strains in single and multiple wards in several hospitals, whereas inter-hospital transmission was relatively uncommon. In summary, we observed significantly genomic diversity of CRKP bacteremia isolates in China, although KPC-2 producing ST11 strains were found to be the most common clonal types. Reducing intra-hospital transmission remains to be the key to control CRKP caused bloodstream infections in China.

**IMPORTANCE** K. pneumoniae is one of the most frequent Gram-negative bacilli isolated from bloodstream infections worldwide and recent studies have shown an increased rate of carbapenem resistance in China. Among carbapenem-resistant K. pneumoniae (CRKP) diverse clones have been reported, especially the high-risk clone ST11, which also exhibited a multidrug resistant phenotype. In addition to the antimicrobial resistance, previous studies have detected strains co-harboring virulent traits, highlighting the potential of transmission of both antimicrobial resistant and virulent strains. Here we studied the antimicrobial resistance profile, virulence genes and hospital transmission of CRKP from bacteremic patients in China. This study showed a high clonal diversity among CRKP, with the predominance of ST11 lineages. We detected virulence markers among multidrug resistant strains, and a high number of genetically similar isolates, suggesting intra-hospital transmission within single and multiple wards. Reducing intra-hospital transmission remains to be the key to control CRKP caused bacteremia in China.

## INTRODUCTION

Klebsiella pneumoniae is the second most frequent Gram-negative bacilli isolated from bloodstream infections worldwide (1). The surveillance antimicrobial resistance program in China reported a high frequency of K. pneumoniae in blood cultures (18.8%) and an overall rate of 5.5% carbapenem resistance in this pathogen during the year 2013 (2). Nonetheless, a recent study in the country reported an increasing prevalence of K. pneumoniae in bloodstream infections from 7% in 2014% to 12% in 2019, and an increasing rate of carbapenem resistance as high as 41.8% (3).

Resistance to carbapenems in K. pneumoniae is mainly mediated by KPC serine-carbapenemase (4, 5), and in a lesser extent by NDM metallo-β-lactamase (MBL) in China (6), which has mainly been reported in isolates recovered in outbreak settings (7). In addition, carbapenem-resistant K. pneumoniae usually exhibits a multidrug resistance profile against most β-lactams, fluoroquinolones and aminoglycosides antibiotics (2), which complicates the selection of adequate treatments and contributes to the overall mortality (8, 9).

Previous studies have detected high genetic diversity in CRKP isolates from bloodstream infections in China, among which common clones include the high-risk ST11strains and ST23, ST15, ST29, ST412 and ST65 (6, 10). Notably, CRKP ST11 was also the predominant clone type among MDR tigecycline-non susceptible K. pneumoniae (11) and extensively drug-resistant (XDR) strains (5) identified in blood cultures in hospitals of China, highlighting its role in the spread of not only carbapenem resistance, but also the resistance to other antibiotics classes.

CRKP dissemination is further obscured by the potential convergence of antimicrobial resistance (AMR) and virulence. Gu et al. (12) reported in 2016 a fatal pneumonia outbreak caused by the acquisition of a virulence plasmid by ST11 CRKP. More recently, a genomic epidemiology study in South and Southeast Asia found 7% of isolates harboring simultaneously important virulence factors such as the aerobactin synthesis locus (*iuc*) and antimicrobial resistance genes (ESBL/carbapenemase). Of major concern, the detection of plasmids carrying both *iuc* and resistance genes suggest the potential co-transfer of this phenotypes (13).

Limited data on both resistance phenotype and genomic characterization of CRKP is reported in bloodstream infections in China. Here we described the genomic characteristics, antimicrobial resistance and main virulence genes in CRKP isolated from patients with bacteremia in 12 hospitals of eight provinces of China.

## RESULTS

### Clinical characteristics of patients with K. pneumoniae bacteriemia.

We analyzed 147 carbapenem-resistant K. pneumoniae (CRKP) isolates from patients with bacteremia in 12 hospitals from eight China provinces between April 2015 and November 2018. Four hospitals covered 65% of the study patients, including hospital H08 (*n* = 39, 26.5%), H04 (*n* = 26, 17.7%), H06 (*n* = 16, 10.9) and H01 (*n* = 15, 10.2%). Patients with bacteremia were predominantly of male sex (*n* = 92, 62.6%) with a median age of 52 years (IQR 39–68 years). These bacteremic patients included 25 infants younger than 1 month of age, from which 22 (88.0%) were diagnosed with early onset bacteremia. The most frequent comorbid conditions diagnosed were gastrointestinal disorders (*n* = 22, 14.9%), cerebral infarction and bleeding (*n* = 17, 11.6%), burns (*n* = 11, 7.5%), respiratory (*n* = 11, 7.5%) and central nervous system infections (*n* = 7, 4.8%). All infant patients with early onset infections were hospitalized in intensive care unit (neonatology and pediatrics), while older patients were mostly hospitalized in intensive care (*n* = 54, 43.6%) or burn units (*n* = 13, 10.5%). The main patient’s clinical characteristics and their hospitalization wards are presented in Table 1.

**TABLE 1:** Clinical characteristics of carbapenem-resistant K. pneumoniae infected patients with bacteremia and their hospitalization wards in 12 referral hospitals in China, 2016–2018

Clinical characteristics/wards^*a*^	ICU (*n* = 55)		Pediatrics neonatology (*n* = 25)		Burn (*n* = 13)		Liver surgery (*n* = 8)		Gastroenterology (*n* = 7)		Neurosurgery (*n* = 7)		Respiratory (*n* = 7)		Neurology (*n* = 6)		Hematology (*n* = 6)		Total (*n* = 147)	
	n	%	n	%	n	%	n	%	n	%	n	%	n	%	n	%	n	%	n	%
Age, yrs (median, IQR)	59	(51–75)	**^b^		45	37–50	46	(43.5–62)	48	(45–63)	55	(40–61)	77	(65–85)	68.5	(53–77)	62	(34–64)	52	(39–68)
Male sex	37	67.2	8	32	8	61.5	4	50	7	100	5	71.4	5	71.4	6	100	4	66.6	92	62.5
Early onset infection	1	1.8	22	88	0	0	0	0	0	0	0	0	0	0	0	0	0	0	23	15.6
Central nervous system infections	7	12.7	0	0	0	0	0	0	0	0	0	0	0	0	0	0	0	0	7	4.7
Coronary heart disease	1	1.8	0	0	0	0	0	0	0	0	0	0	0	0	0	0	0	0	5	3.4
Burns	0	0	0	0	11	84.6	0	0	0	0	0	0	0	0	0	0	0	0	11	7.4
Respiratory conditions	3	5.4	1	4	0	0	0	0	0	0	0	0	3	42.8	0	0	0	0	7	4.7
Cerebral infartaction and hemorrage	8	14.5	0	0	0	0	0	0	0	0	5	71.4	0	0	4	66.6	0	0	17	11.5
Leukemia and cancer	1	1.8	0	0	0	0	3	37.5	0	0	0	0	0	0	0	0	2	33.3	8	5.4
Lung infections	3	5.4	1	4	0	0	0	0	0	0	0	0	4	57.1	0	0	0	0	7	7.4

a only wards with >6 hospitalized patients were shown.

b **, 23 patients were younger than one day (newborns) and two patients were less than 30 days of age.

### Evaluation of antimicrobial susceptibility of K. pneumoniae isolates.

K. pneumoniae isolates showed high resistance (>90%) to all β-lactams, including piperacillin-tazobactam, cefotaxime, ceftazidime, cefepime, aztreonam, imipenem and meropenem (Table 2). Among β-lactams antibiotics, CRKP isolates showed high susceptibility rate to ceftazidime-avibactam (*n* = 110, 74.8%). Resistance to other antibiotics classes was also frequent, with 80.1% (*n* = 119) resistant to ciprofloxacin, 66.0% (*n* = 97) to gentamicin and 51% (*n* = 75) to amikacin. Ninety-eight percent of isolates were resistant to three or more antibiotic classes, and 27.9% were resistant to all six antimicrobial classes evaluated (β-lactams, polymixin, ciprofloxacin, aminoglycosides, tetracyclines and trimethoprim-sulfamethoxazole). Additional susceptibility results are shown in Table 2.

**TABLE 2:** Antimicrobial susceptibility of K. pneumoniae isolated from patients with bacteremia in 12 hospitals in China, 2016–2018

Antibiotics	MIC_50_	%S	%I	%R
Piperacillin-tazobactam	>128	6.8	0.7	92.5
Cefoxitin	>32	1.4	2.0	96.6
Cefotaxime	>64	0.7	1.4	97.9
Ceftazidime	>32	1.4	-^*a*^	98.6
Cefepime	>16	1.4	-	98.6
Aztreonam	>32	4.5	-	92.5
Imipenem	>16	1.4	0.7	97.9
Meropenem	>16	0.0	0.0	100.0
Ceftazidime-Avibactam	2	74.8	-	25.2
Polymyxin B	≤0.5	-	96.6	3.4
Ciprofloxacin	>4	13.6	5.4	80.1
Gentamicin	>16	31.3	2.7	66.0
Amikacin	>64	49.0	-	51.0
Tetracycline	>16	40.1	6.8	53.1
Minocycline	8	17.0	15.0	68.0
Tigecycline	2	73.5	21.1	5.4
Trimethoprim/sulfamethoxazole	>2	45.6	-	54.4

a -, means negative.

### Genetic characteristics of K. pneumoniae isolates.

**FIG 1:**
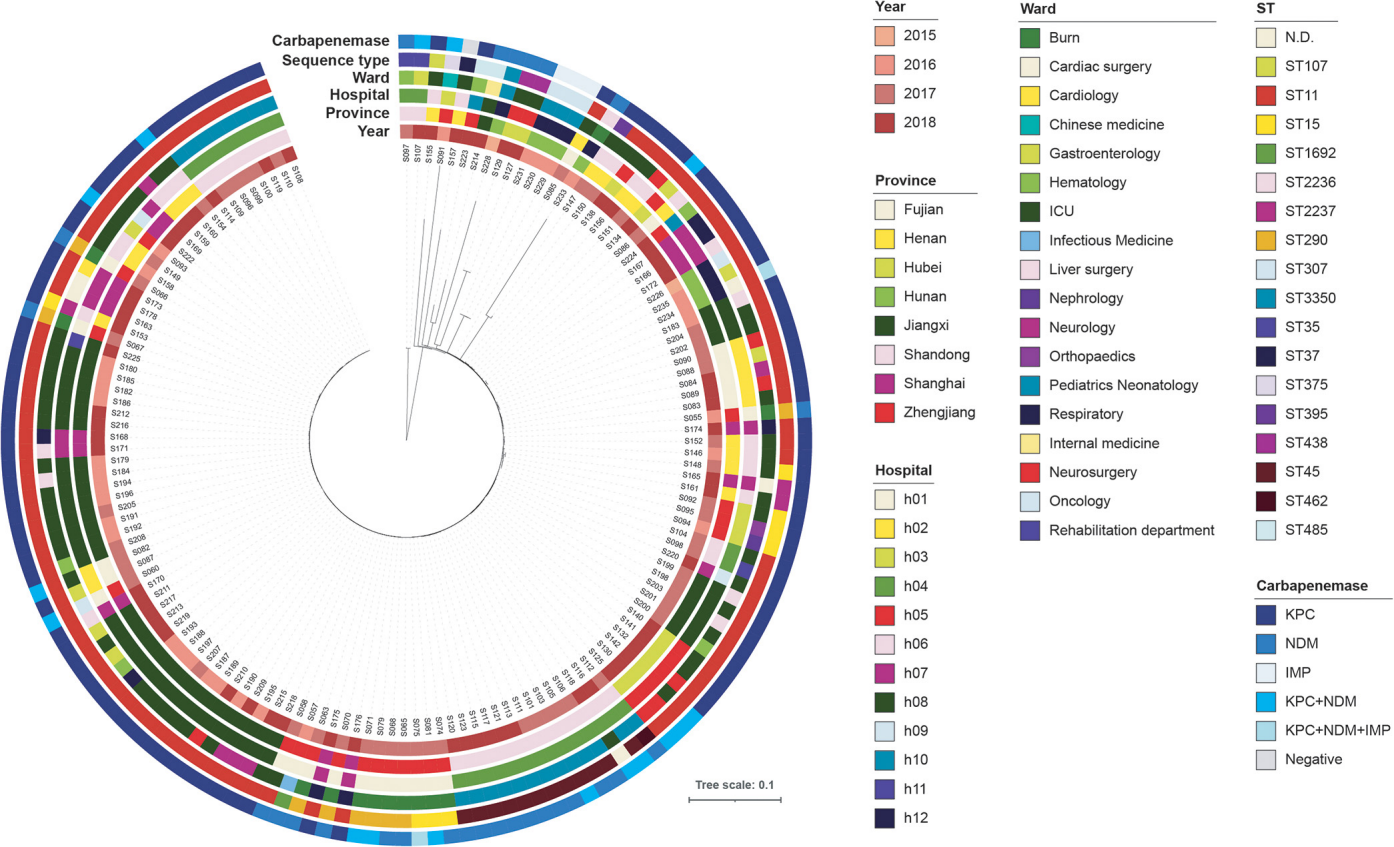
Phylogenetic analysis of 147 genomes of K. pneumoniae causing bacteremia in 12 hospitals from eight China provinces. Colors illustrated province, hospital, ward, sequence type, and type of carbapenemases. Most isolates belonged to sequence type 11 and carried KPC enzymes.

In our study, ST11 accounted for most infections (*n* = 98, 66.6%), followed by ST45 (*n* = 12, 8.2%), ST15 and ST290 (*n* = 8, 5.4% each). Additional STs are shown in Fig. 1. While ST11 was detected in patients from all the participant hospitals, ST45 and ST290 were found only in H04 and H01, respectively. KPC was detected in most isolates (*n* = 98, 66.7%), followed by NDM (*n* = 27, 18.4%), and IMP (*n* = 3, 2.0%), as well as dual-carbapenemases with the combination of KPC, NDM or IMP (Fig. 1, Table 3).

To characterize the virulence genotypes of these CRKP isolates we investigated the capsular polysaccharide type, which is known to increase bacterial survival and dissemination in invasive infections through phagocytosis, complement and antimicrobial peptides inhibition. Among 13 capsular types, we detected the predominance of KL64 (*n* = 75, 51.0%), an emergent capsular in China associated to enhanced virulence and transmissibility (14), followed by KL47 (*n* = 22, 15.0%) and KL24 (*n* = 14, 9.5%). The capsular loci correlated with STs, with KL64 and KL47 exclusively present in ST11, KL24 in ST45, KL19 and KL48 in ST15, and KL21 in ST290 (Table 3).

**TABLE 3:** Selected virulence and antimicrobial resistance characteristic of main STs detected in patients with CRKP bacteremia

Characteristics	ST11 (*n* = 98)	ST15 (*n* = 8)	ST290 (*n* = 9)	ST45 (*n* = 12)	Others (*n* = 20)	Total (*n* = 147)
Capsule locus						
KL19	-^*a*^	4 (50.0)	-	-	2 (10.0)	6 (4.1)
KL21	-	-	9 (100.0)	-	-	9 (6.1)
KL24	-	-	-	12 (100.0)	2 (10.0)	14 (9.5)
KL47	22 (22.5)	-	-	-	-	22 (15.0)
KL48	-	4 (50.0)	-	-	-	4 (2.7)
KL64	75 (76.5)	-	-	-	-	75 (51.0)
Other	1 (1.0)	-	-	-	16 (80.0)	17 (11.6)
Yersiniabactin						
Negative	1 (1.0)	5 (62.5)	6 (66.7)	-	12 (60.0)	24 (16.3)
*ybt* 4 – plasmid	-	-	-	-	3 (15.0)	3 (2.0)
*ybt 9* – ICEKp3	97 (99.0)	-	1 (11.1)	-	-	98 (66.7)
*ybt* 10 – ICEKp4	-	-	-	12 (100.0)	1 (5.0)	13 (8.8)
*ybt* 14 – ICEKp5	-	3 (37.5)	-	-	2 (10.0)	5 (3.4)
*ybt* 15 – ICEKp11	-	-	-	-	1 (5.0)	1 (0.7)
*ybt* 16 – ICEKp12	-	-	-	-	1 (5.0)	1 (0.7)
*ybt* (unknown)	-	-	2 (22.2)	-	-	2 (1.4)
Aerobactin (lineages)						
iuc1	52 (53.1)	-	-	1 (8.3)	6 (30.0)	59 (40.1)
iuc3	1 (1.0)	2 (25.0)	9 (100.0)	-	-	12 (8.16)
iuc unknown	1 (1.0)	-	-	-	-	1 (0.7)
Negative	44 (44.9)	6 (75.0)	-	11 (91.7)	14 (70.0)	75 (51.0)
Hypermucoid (RmpADC)						
rmp1-KpVP1	31 (31.6)	-	-	-	2 (10.0)	33 (22.45)
rmp (unknown)	3 (3.1)	-	-	-	1 (5.0)	2 (2.7)
Negative	64 (65.31)	8 (100.0)	9 (100.0)	12 (100.0)	17 (85.0)	110 (74.8)
Carbapenemases						
KPC	88 (89.8)	5 (62.5)	-	-	5 (25.0)	98 (66.7)
NDM	1 (1.0)	1 (12.5)	7 (77.8)	10 (83.3)	8 (40.0)	27 (18.4)
IMP	-	-	-	-	3 (15.0)	3 (2.0)
KPC+NDM	8 (8.2)	1 (12.5)	2 (22.2)	2 (16.7)	3 (15.0)	16 (10.9)
KPC+NDM+IMP	1 (1.0)	1 (12.5)	-	-	-	2 (1.36)
Negative					1 (5.0)	1 (0.7)
OmpK mutations						
OmpK35 truncated	3 (3.1)	-	-	-	5 (25.0)	8 (5.4)
OmpK36 truncated		1 (12.5)		1 (8.3)	1 (5.0)	3 (2.0)
OmpK36GD and OmpK35 truncated	92 (93.9)	-	-	-	-	92 (62.6)
OmpK35 and OmpK36 truncated	3 (3.1)	-	1 (11.1)	-	2 (10.0)	6 (4.1)
Negative	-	7 (87.5)	8 (88.9)	11 (91.67)	12 (60.0)	38 (25.9)

a -, means negative.

We also analyzed additional key virulence factors in K. pneumoniae such as yersiniabactin, aerobactin and salmochelin siderophore systems, which enhance bacterial survival through iron acquisition from host proteins. We detected yersiniabactin in most isolates (*n* = 123, 83.7%), mainly from ST11, ST15, ST290 and ST45 genetic backgrounds. We also found that yersiniabactin alleles were linked to two main STs. ST11 carried *ybt9* in ICEKp3 (97/98), while ST45 carried *ybt10* in ICEKp4 (12/13). Aerobactin was also detected in 49.9% of isolates (*iuc1* and *iuc3*), and 42.2% (*n* = 61) of isolates carried both yersiniabactin and aerobactin simultaneously. All isolates were negative for colibactin, but eight carried salmochelin (*n* = 8, 5.4%), seven of which were ST11 (Table 3).

In addition, we investigated the presence of genes *rmpADC* and *rmpA2* associated to the hypermucoviscous phenotype in K. pneumoniae isolates. *rmpA* act as a transcriptional regulator for the *rmpC* involved in upregulation of capsule expression (15) and *rmpD* is a transmembrane protein essential for the hypermucoviscosity phenotype (16). The *rmpADC* locus was present in 25.2% (*n* = 37) of isolates and 89.2% (*n* = 33) of positive isolates belonged to allele *rmp1* which is associated to virulence plasmid KP-VP-1 (Table 3). All but two isolates *rmp1* positive belonged to ST11 (*n* = 31, 93.4%).

As antimicrobial resistance could vary depending on patient’s age, we also explored distribution of drug resistance genes among different age groups (<1 month, from 1 month to 1 year, 1-19 years, 20-39 years, 40-59 years, 60-79 years, >80 years). We found that gene distribution was similar among age categories, except for NDM-1 which was more frequent among children <1 month infected by ST45.

### Antimicrobial resistance genes in K. pneumoniae isolates.

We also examined the genes associated with carbapenem resistance as well as the multidrug resistant pattern observed in the phenotypic susceptibility testing. Analysis of antimicrobial resistant genes demonstrated acquired carbapenemases genes in 146 isolates, which predominantly harbored *bla*_KPC-2_ (77.9%), *bla*_NDM-1_ (14.5%), *bla*_NDM-5_ (13.8%), and *bla*_IMP-38_ (2.1%). In addition, 87.1% isolates carried ESBL genes with the predominance of *bla*_CTX-M-65_ (65.6%) and *bla*_CTX-M-15_ (18.9%). Notably, we detected *ompK35* or *ompK36* mutations in 74.2% (*n* = 109) isolates (Table 3). Among these mutations the most frequent was OmpK36GD (*n* = 92, 66.1%), which consist of Gly134Asp135 duplication in loop 3 of the OmpK36 porin. This mutation constricts the pore channel, restricting diffusion of both carbapenems and nutrients. All isolates carrying this mutation belonged to ST11.

Resistance to aminoglycoside class antibiotics was mediated by aminoglycoside transferases, including adenylyl-transferase *aadA2* (57.8%) and *aadA16* (21.1%), phosphotransferases *aph(3″)-Ib* (42.4%), *aph(*6*)-Id* (41.1%), and acetyltransferase *aac(*3*)-IId* (34.7%). We also detected several mutations in the quinolone resistance determinant region (QRDR) in *gyrA* and *parC* genes in 72.2% isolates, and *qnrS1* gene associated with plasmid mediated quinolone resistance in 42.2% of isolates. We also detected genes associated with tetracycline resistance in 55.1% isolates (*tet(A) and tet(D)*), trimethoprim resistance in 57.8% isolates (*dfrA*) and sulfonamides resistance mediated by *sul1* (74.2%) and *sul2* (50.3%). We only detected one isolate harboring *mcr-1* gene which had a high MIC to polymixins (16 μg/mL). Analysis of drug-resistance gene in isolates with different capsular loci showed no relationship among gene composition and capsular types (data not shown).

### Klebsiella pneumoniae dominant clones and potential hospital transmission.

We then focused on two STs (ST11 and ST45) with > 10 isolates among patients with bacteremia to probe potential intra- and inter- hospital transmissions.

**FIG 2:**
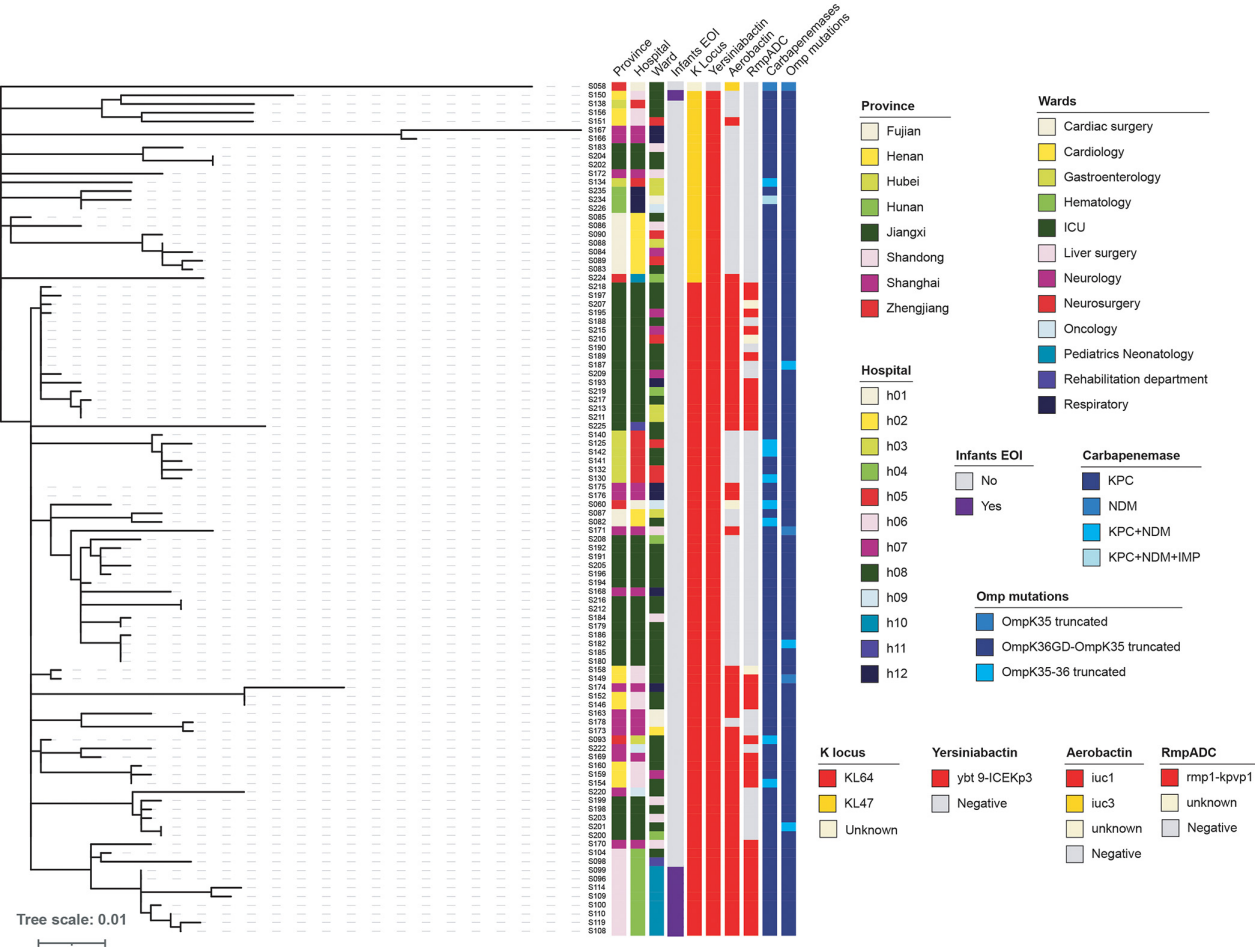
Phylogenetic analysis of 98 K. pneumoniae ST11 genomes causing bacteremia in hospitals from China. Colors illustrated province, hospital, ward, early onset bacteremia in infants, capsular locus, yersiniabactin, aerobactin, hypermucoid loci (r*mpADC*), type of carbapenemase and Omp mutations.

**ST11.** CRKP ST11 was identified in all the participant hospitals. Two main subclones were identified among ST11 population (*n* = 98), one with capsular locus 47 (ST11-KL47) (22/98) and another with capsular locus 64 (ST11-KL64) (75/98). ST11-KL64 was detected in 10/12 hospitals and was frequently identified in ICU of hospital 08 (20/35), followed by Pediatrics unit of hospital 04 (8/10). In contrast, ST11-KL47 was detected in 7/12 hospitals causing infections in multiple wards. Both clones were detected in patients with multiple comorbidities (Fig. 2).

Although ST11 clone predominated in all study hospitals, we identified closely related ST11 isolates (<21 SNPs) linked to unique hospitals or between hospitals suggesting both intra-hospital and inter-hospital transmission. Of note, in hospital 08 we detected intra-hospital transmission of three ST11-KL64 sub-clusters with different profiles of virulence factors. The largest cluster included 16 isolates recovered from multiple wards between 2016 and 2018, encoding yersiniabactin, aerobactin and RmpADC. The second cluster included 14 isolates encoding only yersiniabactin mainly recovered from ICU in 2016. Closely related isolates were also detected in hospital h01, h02 and h07. The third cluster included five isolates encoding both yersiniabactin and aerobactin collected in ICU, surgery and gastroenterology departments in 2017. All isolates in these clusters were KPC producers, and carried OmpK36GD mutations and Omp35 truncation (Fig. 2).

Additional hospital associated ST11 strains were detected in hospitals h02, h04 and h05 (Fig. 2). In particular, in hospital 04 of Shandong province we detected a cluster of 10 ST11-KL64 isolates encoding the virulence factors yersiniabactin, aerobactin and RmpADC and KPC carbapenemase. Eight of them were recovered from infants diagnosed with early onset bacteremia hospitalized in the pediatric department in 2017 and 2018. In addition, in hospital h02 from Fujian province we detected ST11- KL47 causing infections in multiple wards (ICU, surgery, gastroenterology and neurology) during 2017–2018. This clone was yersiniabactin-positive, aerobactin-negative and RmpADC-negative, KPC-positive and carried mutations in porin genes (OmpK36GD and Omp35 truncated). Similarly, in 2018 in hospital h05 from Hubei province we detected transmission of ST11-KL64 in neurosurgery and ICU of a yersiniabactin-positive clone harboring KPC and NDM enzymes and Omp mutations (Fig. 2).

**ST45.** We detected intra-hospital transmission of ST45 isolates in the neonatology and pediatrics unit of hospital 04 in the province of Shandong (Fig. 1). We detected 12 cases with early onset bacteremia from May 2017 to August 2018 caused by ST45 clone. The remaining cases of bacteremia in children younger than 1 month were caused by a small number of diverse clones (ST11, ST307, ST3350 and ST462). All blood cultures in ST45 infected children were taken within an hour of birth and were positive for K. pneumoniae, suggesting vertical transmission of hospital acquired strains from mother to child or environmental contamination. All isolates carried capsular locus (KL24), and were positive for yersiniabactin (lineage 10) but negative for salmochelin and *rmpA*. In addition, all isolates were positive for NDM-1, and two isolates also carried KPC-2. ST45 clone was resistant to all β-lactams tested, including imipenem and meropenem, and only four isolates were susceptible to ceftazidime-avibactam. However, this clone exhibited susceptibility to amikacin (100%, *n* = 12) and ciprofloxacin (91.7%, *n* = 11).

## DISCUSSION

Our multi-center study showed a high diversity of STs (>17 STs, Fig. 1) causing bloodstream infections, with ST11 accounting for 66.6% of isolates. Importantly, we found 75% of isolates were involved in intra-hospital transmission. We detected transmission of hospital-specific ST11 subclones encoding virulence factors, and the nosocomial spread of ST45, ST290 and ST15 strains. Each clone exhibited variations in antimicrobial resistance and virulence profile, such as the production of KPC-2 by ST11 and ST15, and NDM-1 and NDM-5 by ST45 and ST290, respectively.

In this study, the majority of BSI CRKP isolates were recovered from patients hospitalized in ICU, neonatology and burn units. Patients hospitalized in these departments are at greater risk of CRKP infections mainly because immune status alteration (17, 18), use of broad-spectrum antibiotics (17, 19), frequent comorbidities (17, 18), and use of invasive devices (19, 20). Among pediatric patients, we detected several cases of early onset bacteremia caused by K. pneumoniae ST11 and ST45. In our study, early onset infection caused by CRKP presented in premature neonates, which is one of the main risk factors for infection caused by MDR-GN bacteria (21). Additional patient and center level risk factors for MDR infection include maternal or neonatal MDR colonization, invasive devices, history of prior unit outbreak, suboptimal infection control practices, and high antibiotic consumption (22). In hospitals where CRKP transmission was detected, maternal and infant screening for CRE carriage could lead to early detection and implementation of eradication measures to prevent new cases of transmission.

Besides carbapenem resistance, bacteremia isolates encoded a number of virulence genes associated to invasive infections. Previous study showed siderophore systems such as yersiniabactin, aerobactin, colibactin, salmochelin and regulators of hypermucoid phenotype *rmpA* are more frequently found in invasive than non-invasive isolates. While yersiniabactin is reported in nearly 30% of non-invasive isolates (23), we detected it in 83.7% of bacteremia isolates. Similarly, aerobactin was detected in 10% of non-invasive isolates (23), while in this study it was present in 49.0% of isolates. We also detected a higher frequency of *rmpA* in bacteremia isolates (22.5%) compared to non-invasive isolates in a previous study (less than 10%) (23).

We found a high clonal diversity (>17 STs) among 147 CRKP K. pneumoniae bloodstream infection isolates. Forty-four percent of isolates belonged to ST15, ST45, ST35, ST290, ST307, ST438, ST485, and single isolates of ST107, ST1692, ST2236, ST3350, ST37, ST375, ST395, ST462 were detected. Previous work also showed 57.4% of CRKP isolated from different type of infections (*n* = 140) belonged to 15 different STs in the Jiangxi Province in China, with ST11 being the most dominant (24). Importantly, genomic analysis has shown heterogeneity within ST11 clone in China based on core SNP analysis that correlated with *cps* loci, being KL64 and KL47 two main sublineages (25). We also detected specific sublineages of ST11-KL64 and ST11-KL47 strains spreading in different hospitals. Among these strains, KPC-2 producing ST11-KL64 strains encoding virulence factors such as yersiniabactin, aerobactin and RmpADC were detected in six hospitals. In two of six hospitals, a cluster of at least 10 patients affected by this clone was identified. Similar ST11-KL64 strains have been detected in China associated to fatal hospital outbreaks (12), ICU dissemination (26) and severe infections (27). These hypervirulent strains carried a pLVKP-like plasmid encoding the virulence factors *rmpA* and aerobactin (12, 27). The facts that previous data indicate MDR K. pneumoniae clones are likely to acquire virulence genes (28), and that they can adapt to ameliorate the fitness cost of plasmid acquisition (29), highlight the risk of the convergence of multidrug resistance and virulence, which seem to be common phenomenon in K. pneumoniae BSI in southeast Asia (13, 30). Similar to the CRKP epidemics in Europe (31), it appears that CRKP BSI in China is driven by hospital spread of ST11 specific lineages within hospitals, stressing the role of genomic surveillance to identify the high-risk clones during its early expansion.

We also detected intra-hospital transmission of NDM-producing ST45 in newborns patients in one hospital. ST45 have been reported previously causing bacteremia in adults (32, 33), but also infections in pediatrics populations (34–36). Previous study showed GES-5 producing ST45 clone displayed resistance to all carbapenems and encoded the genes associated to K type antigen KL24 (35), similar to our study. Although resistant to carbapenems, ST45 was susceptible to other antibiotic classes, including aminoglycosides and ciprofloxacin. In contrast to other clones, ST45 isolates were resistant to ceftazidime-avibactam because of the NDM production.

ST15 and ST290 CRKP clones were detected in a less frequency in our study. KPC producing ST15 K. pneumoniae have been found causing outbreaks (37), and is the second most frequent CRKP clone in hospital infections after ST11 (38, 39). However in a recent surveillance study conducted in one hospital in northeast China, Chen et al. (40), reported a shift in dominance from ST11 to ST15 during the year 2020 compared to the trend observed between 2015 to 2019. Susceptibility to amikacin was a prominent characteristic of ST15 isolates, while resistance to this antibiotic was reported in ST11 isolates in both the previous and our study. Interestingly, additional studies have reported an increasing detection of ST290. A previous surveillance study conducted in Shanghai (41), reported the presence of ST290-KL21 clone encoding KPC-2 in a tertiary hospital in China. Later in 2016–2017 an outbreak of ST290 harboring NDM-5 was reported in a burn unit of one hospital in east China (7). These studies seem to indicate a changing distribution in the predominance of ST11 in favor of additional STs in the recent years in some China hospitals.

Our results showed the predominance of KPC enzymes among CRKP isolates, and additional MBLs such as NDM were also increasingly detected. These K. pneumoniae isolates showed >90% resistance to almost all antibiotics evaluated in the β-lactam classes. Although ceftazidime-avibactam is considered a good alternative for treating infections caused by CRKP, we found resistance to this antibiotic in 25.2% (*n* = 37) isolates. The majority of these isolates carried NDM (29/37, 78.4%) and three additional isolates carried IMP enzymes which could be related to ceftazidime-avibactam resistance. Despite of the presence of MBLs, our results still suggested ceftazidime-avibactam is one of the best candidates for antibiotic treatment in patients with CRKP (especially for KPC-producing) bacteremia in our study population. Surveillance of AMR trends should be maintained because of the acquisition of MBLs in different clones and the potential emergence of resistance to ceftazidime-avibactam mediated by amino acid changes in KPC enzyme as previously described (42).

Resistance to other antibiotic classes including aminoglycosides and fluoroquinolones was >80% in CRKP isolates. As expected, several genes encoding aminoglycoside modifying enzymes were detected in the genomic analyses, among them acetyltransferases (*aac3*-IId, 40.4%), adenyltransferases (*aadA2*, 67.5%) and phosphotransferases (*aph*3-Ib, 49.2%) were the most common. Resistance to ciprofloxacin appeared to be mediated mainly by *gyrA* mutations (D87G, 76.4%, and S83I, 80.3%). In addition, resistance to polymyxin B was detected in five isolates, of which one harbored the plasmid gene *mcr-1.1* and two contained *mgrB* mutations (truncation and G37S mutation, respectively). The polymyxin B resistance mechanisms in other two stains remained to be determined. Of note, our results showed 73.5% CRKP isolates susceptible to tigecycline (MIC lower than 2 mg/L). We found a higher tigecycline susceptibility than the results from previous work where CRKP demonstrated a susceptibility rate of 40.2% in 25 tertiary hospitals in 14 provinces of China (43). However, the use of tigecycline in endovascular infections such as bacteremia is still controversial due to poor serum concentration.

Our study has some limitations. First, the selection of isolates was done according to its availability in the microbiology laboratory, which can bias the analysis of the burden of different clones in hospital dissemination. In addition, the inaccessibility of patient’s information about mortality and additional characteristics made unfeasible the analysis of the role of clinical, genetic and antimicrobial resistance determinants on patient mortality. Finally, we did not phenotypically evaluate the virulence potential of K. pneumoniae clones encoding virulence traits.

## CONCLUSIONS

In this study, we detected intra-hospital transmission of ST11 specific sub-lineages within several hospitals. Importantly, ST11 clone showed a multidrug resistant phenotype to routine antimicrobials used to treat K. pneumoniae infections and we found a high susceptibility to ceftazidime-avibactam suggesting its efficacy for treating CRKP infections. The frequent detection of yersiniabactin, *rmpA* and aerobactin among CRKP ST11-KL64 alert over the potential convergence of antimicrobial resistance and virulence into the same genetic background and its spread in hospitals settings. Alarmingly, ∼75% of isolates were associated with intra-hospital transmission, and consequently, reducing intra-hospital transmission remains to be the key to control CRKP bacteremia in China.

## MATERIALS AND METHODS

### Study design.

We retrospectively characterized 147 unique (one isolate per patient) carbapenem-resistant K. pneumoniae isolates from patients with bacteremia attended in 12 referral hospitals in China between April 2015 and November 2018. Hospitals were located in Fujian, Henan, Hubei, Hunan, Jiangxi, Shandong, Shanghai and Zhengjiang provinces in China. Study hospitals were selected from east and south-central provinces of China with the highest carbapenem-resistant rate according to the 2014 China Antimicrobial Resistance Surveillance Report. We selected in each provinces the major referral hospitals where complex infections such as bacteremia are diagnosed and treated.

CRKP bacteremia was defined as the CRKP isolation in at least one positive blood culture. CRKP was defined as isolates intermediate or resistant to imipenem or meropenem (MICs ≥ 2 μg/mL). Basic clinical characteristics of patients were obtained from clinical charts, including age, sex, ward, date of isolation, comorbidities and early onset bacteremia. Early onset bacteremia was defined as bacteremia occurring in the first 3 days of life in preterm infants or 7 days in term infants. Late onset bacteremia is the infection occurring after three and 7 days in preterm and term infants, respectively, up to 120 days (44). We conducted a phenotypic susceptibility testing and whole genome sequence analysis to characterize antimicrobial resistance profile and virulence genes of CRKP clones recovered from these patients with bacteremia.

### Susceptibility evaluation.

Hospital laboratories performed bacterial identification and antimicrobial susceptibility testing using VITEK2 system. MICs were measured for cefoxitin, cefotaxime, aztreonam, cefepime, ceftazidime, piperacillin-tazobactam, imipenem, meropenem, gentamicin, amikacin, ciprofloxacin, tetracycline, minocycline and trimethoprim-sulfamethoxazole. MICs to ceftazidime-avibactam, tigecycline and polymyxin B were tested by broth microdilution. The testing was performed in duplicate on two different days. Quality control (QC) strains Pseudomonas aeruginosa ATCC 700603 and Escherichia coli ATCC 25922 were used in all testing. Clinical breakpoints were interpreted according to Clinical and Laboratory Standards Institute 2021 guidelines (45), except for tigecycline, which was interpreted according to the U.S. Food and & Drug Administration (FDA) criteria (https://www.accessdata.fda.gov/drugsatfda_docs/label/2013/021821s026s031lbl.pdf).

### Whole genome sequencing analysis.

Genome sequencing was carried out using the HiSeq 2500 sequencing platform (Illumina Inc., San Diego, CA), with 2 × 150 bp paired-end reads. Raw data were filtered with Trimmomatic v0.39 (46), followed by assembly using Spades v3.14 (47). Kleborate v2.0 was used to screen genome assemblies for MLST, capsular type and virulence loci such as siderophore systems yersiniabactin (*ybt*), aerobactin (*iuc*), colibactin (*clb*), and salmochelin (*iro*), and hypermucoid loci *rmpADC/rmpA2* (48). Filtered reads from each isolate were mapped to K. pneumoniae ST11 reference genome (strain HS11286, Accession number CP003200) by Snippy 4.4 (https://github.com/tseemann/snippy) using default settings, followed by removing SNPs at the repeated, prophage and recombination regions. Core SNP tree was constructed using the method described previously (49). In this study, we used the previously defined 21 SNPs as a threshold to discriminate hospital clusters in K. pneumoniae, given this value minimize the number of false positives and false negative pairs to identify hospital transmission (31). The raw reads of the 147 genomes sequenced in this study were deposited in GenBank bioproject accession no. PRJNA354234.

### Statistical analysis.

We performed a descriptive analysis to analyze the clinical and molecular characteristics of CRKP isolates causing bacteremia. Categorical data was summarized using absolute and relative frequencies, and the distribution of non-normal quantitative data was summarized using the median and interquartile range. All statistical analysis was performed in STATA/IC 15.1 (50).

### Data availability.

The data sets analyzed in this study can be found in online repositories. The names of the repository/repositories and accession number(s) can be found in the article.
